# Intermittent Hypoxic Conditioning Alleviates Post-Traumatic Stress Disorder-Induced Damage and Dysfunction of Rat Visceral Organs and Brain

**DOI:** 10.3390/ijms21010345

**Published:** 2020-01-05

**Authors:** Eugenia B. Manukhina, Vadim E. Tseilikman, Marina N. Karpenko, Nina S. Pestereva, Olga B. Tseilikman, Maria V. Komelkova, Marina V. Kondashevskaya, Anna V. Goryacheva, Maxim S. Lapshin, Pavel O. Platkovskii, Alexey P. Sarapultsev, Anatoly V. Alliluev, H. Fred Downey

**Affiliations:** 1 School of Medical Biology, South Ural State University, Chelyabinsk 454080, Russia; 2 Laboratory for Regulatory Mechanisms of Stress and Adaptation, Institute of General Pathology and Pathophysiology, Moscow 125315, Russia; 3 Department of Physiology and Anatomy, University of North Texas Health Science Center, Fort Worth, TX 76107, USA; 4 I.P. Pavlov Physiology Department, Institute of Experimental Medicine, St. Petersburg 197376, Russia; 5 School of Basic Medicine, Chelyabinsk State University, Chelyabinsk 454001, Russia; 6 Laboratory for Immunomorphology of Inflammation, Research Institute of Human Morphology, Moscow 117418, Russia; 7 Laboratory of Immunopathophysiology, Institute of Immunology and Physiology of the Ural Branch of the Russian Academy of Sciences, Ekaterinburg 620049, Russia

**Keywords:** posttraumatic stress disorder, intermittent hypoxia conditioning, heart, liver, brain, oxidative stress

## Abstract

Posttraumatic stress disorder (PTSD) causes mental and somatic diseases. Intermittent hypoxic conditioning (IHC) has cardio-, vaso-, and neuroprotective effects and alleviates experimental PTSD. IHC’s ability to alleviate harmful PTSD effects on rat heart, liver, and brain was examined. PTSD was induced by 10-day exposure to cat urine scent (PTSD rats). Some rats were then adapted to 14-day IHC (PTSD+IHC rats), while PTSD and untreated control rats were cage rested. PTSD rats had a higher anxiety index (AI, X-maze test), than control or PTSD+IHC rats. This higher AI was associated with reduced glycogen content and histological signs of metabolic and hypoxic damage and of impaired contractility. The livers of PTSD rats had reduced glycogen content. Liver and blood alanine and aspartate aminotransferase activities of PTSD rats were significantly increased. PTSD rats had increased norepinephrine concentration and decreased monoamine oxidase A activity in cerebral cortex. The PTSD-induced elevation of carbonylated proteins and lipid peroxidation products in these organs reflects oxidative stress, a known cause of organ pathology. IHC alleviated PTSD-induced metabolic and structural injury and reduced oxidative stress. Therefore, IHC is a promising preventive treatment for PTSD-related morphological and functional damage to organs, due, in part, to IHC’s reduction of oxidative stress.

## 1. Introduction

Posttraumatic stress disorder (PTSD) is a condition caused by severe stress and subsequent chronic distress, which is generally associated with a life-threatening experience, such as a natural disaster, military combat, traffic accidents, incurable disease or a personal tragedy. PTSD may result in neuropsychological [1,2], cardiovascular [3], gastrointestinal, metabolic, endocrine, and even oncological diseases [4]. Current pharmacological treatment for PTSD is not adequately effective and may cause serious side effects.

Although incidence of PTSD is increasing, approximately 60–80% of humans and animals exposed to severe stress do not develop PTSD [5,6,7]. This individual resistance to PTSD is apparently based on genetically determined potency of endogenous defense systems also called stress-limiting systems, which include heat shock proteins (HSPs), antioxidants, nitric oxide, prostaglandins, and serotonergic, GABAergic and other systems [8,9,10].

Endogenous defense systems can be enhanced by intermittent hypoxia conditioning (IHC), which has been shown to be efficient, safe, and free of side effects [8]. In particular, IHC is highly cardio-, vaso-, and neuroprotective [11,12]. In clinical and experimental studies, IHC prevented behavioral disorders and cerebral neuronal damage in epilepsy [13], Parkinson disease [14], and Alzheimer’s disease [12,15]. IHC preconditioning [16] and postconditioning [17] were shown to abolish development of stress-induced anxiety in the rat “stress-restress” model of PTSD. Recently we found that rats treated with IHC prior to predator stress exhibited significantly less anxiety-like behavior, a lesser drop of plasma corticosterone, and reduced structural signs of adrenal gland dystrophy, all signs characteristic of PTSD [18]. In fact, IHC exerted a robust anti-stress effect in naïve rats, as evidenced from less anxiety during an elevated X-maze test [18].

## 2. Results

### 2.1. IHC Alleviated Behavioral Markers of PTSD

Experimental PTSD increased the time spent in the closed arms of the elevated X-maize (Table 1) by 8.4% compared to the control group (*p* < 0.001), while IHC alone reduced this time by 10% (*p* < 0.001). For the PTSD+IHC group, the time spent in the closed arms was 11% shorter than for the PTSD group (*p* < 0.001), and this time did not significantly differ from that of the control group. Therefore, IHC prevented the PTSD-induced increase in the time spent in closed arms of the X-maize.

Experimental PTSD decreased the time spent in open arms by 42% (*p* = 0.012) whereas IHC increased this index two times (*p* = 0.015) compared to the control. In the PTSD+IHC group, the time spent in open arms was 74% longer than in the PTSD group (*p* < 0.001) and did not significantly differ from the control. Therefore, IHC abolished the PTSD-induced decrease in the time spent in open arms. 

In PTSD rats, AI was 9.7% higher than in control rats (*p* = 0.035). IHC alone did not significantly change the AI value (*p* = 0.31); however, in the PTSD+IHC group, AI was 7% lower than in PTSD (*p* = 0.042) and did not significantly differ from the control value. Therefore, IHC prevented the PTSD-induced increase in AI, which was consistent with the effect of IHC on the changes in time spent in open and closed arms of the X-maze.

### 2.2. IHC Prevented Detrimental Effects of PTSD on the Heart

#### 2.2.1. IHC Prevented PTSD-Mediated Decrease in Myocardial Glycogen

**Figure 1:**
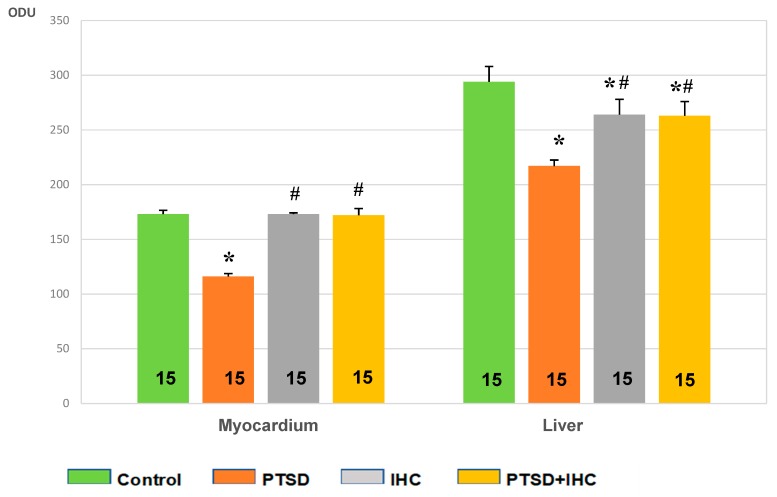
Effect of PTSD, IHC, and PTSD+IHC on glycogen content in the rat heart and liver. ODU = optical density units. PTSD = posttraumatic stress disorder; IHC = intermittent hypoxic conditioning. Data are presented as means ± SEM. Number of rats in groups is shown on the bars. * Significantly different from control; # significantly different from PTSD. Specific *p* values are stated in the text.

PTSD resulted in a 33% decrease in myocardial glycogen compared to the control (*p* < 0.001) (Figure 1). IHC alone did not induce any change in glycogen. In stressed rats, IHC completely prevented the stress-induced exhaustion of myocardial glycogen; in the PTSD+IHC group, the myocardial content of glycogen was 33% higher than in the PTSD group (*p* < 0.001) and did not significantly differ from the control.

**Figure 2:**
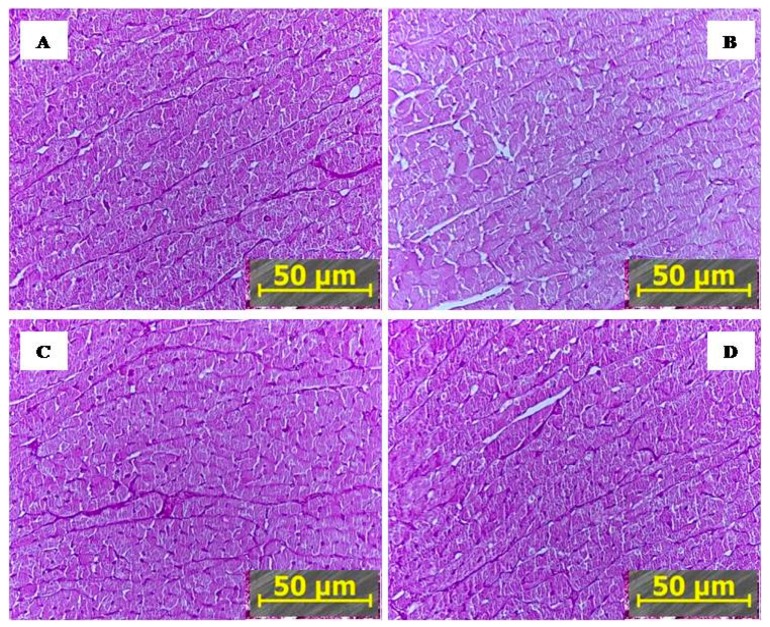
Representative microscopy of glycogen in the rat heart, cross sections (Chic reaction). (**A**) control, (**B**) PTSD; (**C**) IHC, (**D**) PTSD+IHC. Arrows indicate cells deprived of glycogen. Magnification ×200.

Figure 2 shows that staining of glycogen in sections of heart tissue from PTSD rats (Figure 2B) was considerably less intensive than in the control (Figure 2A). The glycogen reaction was similar in liver of control (Figure 2A), IHC (Figure 2C), and PTSD+IHC rats (Figure 2D) and significantly more intensive than in the liver of PTSD rats (Figure 2B).

#### 2.2.2. IHC Markedly Reduced PTS-Mediated Myocardial Damage

In hearts from 15 untreated control rats, histological analysis found no morphological damage. Eight hearts of 15 PTSD rats had myocardial damage (*p* < 0.005 vs. control rats). Only one heart of 15 IHC rats had damage (*p* > 0.05 vs. control rats). Two hearts of 15 IHC+PTSD rats had damage (*p* < 0.05 vs. PTSD rats and *p* > 0.05 vs. control rats).

**Figure 3:**
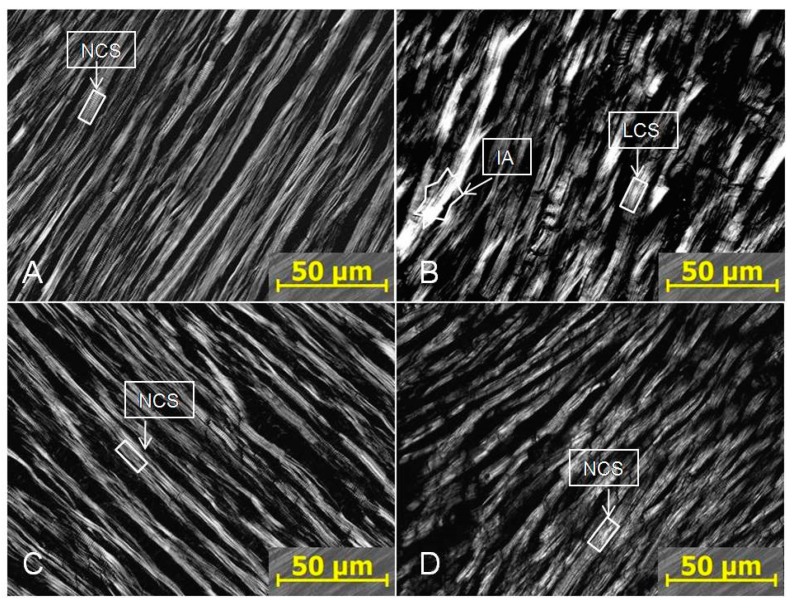
Polarizing microscopy of the rat myocardium. (**A**) Control; (**B**) PTSD; (**C**) IHC; (**D**) PTSD+IHC. Magnification ×400. NCS = normal cross-striation; LCS = loss of cross-striation; IA—ischemic area.

The specific myocardial changes caused by PTSD are shown in Figure 3. Normal cardiomyocytes observed in the heart from control, IHC, and PTSD+IHC rats had clear cell borders and cross-striation (Figure 3A,C,D). In the myocardium of PTSD rats, cell borders were often blurred, and cross striations were lost at some sites. These findings reflected focal alterations in sarcomere structure due mostly to I-disk destruction. In some areas, A disks merged into a solid, glowing, white conglomerate. These changes suggest the presence of segmental metabolic and hypoxic injuries and impaired myocardial contractility [19]. Ischemic area with focal disaggregation and lysis of myofibrils were visible in the myocardium of PTSD rats (Figure 3B). Since signs of myocardial damage in PTSD+IHC rats were rare, these results demonstrated a remarkable ability of IHC to ameliorate PTSD-induced myocardial injury.

#### 2.2.3. IHC Alleviated Oxidative Stress Induced by PTSD in the Heart

**Figure 4:**
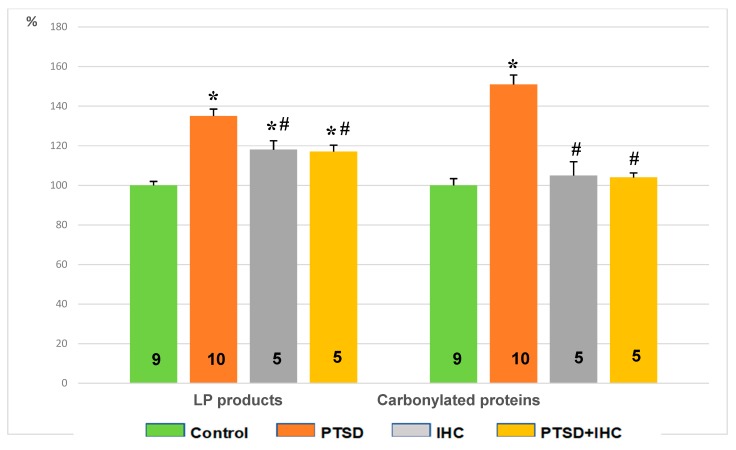
Effects of PTSD, IHC, and PTSD+IHC on concentrations of oxidative stress markers in the rat myocardium. PTSD = posttraumatic stress disorder; IHC = intermittent hypoxic conditioning; LP = lipid peroxidation. Data are presented as means ± SEM in percent of control values (for control, LP products = 0.396 ± 0.024 oxidation conventional units; carbonylated proteins = 408 ± 42 oxidation conventional units). Numbers of rats in the groups are shown on the bars. * Significantly different from control; ^#^ significantly different from PTSD. Specific *p* values are stated in the text.

Figure 4 shows that PTSD increased LP and protein oxidation products by 35% and 50%, respectively, compared to control (*p* < 0.001 for both). IHC alone increased LP by 18% compared to control (*p* = 0.004) and left unchanged the content of protein carbonyls. At the same time, IHC significantly limited oxidative stress in the myocardium, which was evident by a 14% decrease in LP products and 23% decrease in protein oxidation products in the PTSD+IHC group compared to the PTSD group (*p* = 0.002 for both). However, the indexes of oxidative stress remained significantly higher than in the control group.

### 2.3. IHC Prevented Detrimental Effects of PTSD on the Liver

#### 2.3.1. IHC Reduced PTSD-Mediated Depletion of Hepatic Glycogen

Figure 1 shows that PTSD rats had a 26% decrease in hepatic glycogen compared to control (*p* < 0.001). IHC alone moderately reduced hepatic glycogen (*p* < 0.05) but significantly alleviated the stress-induced decrease in the glycogen content (*p* < 0.05). PTSD+IHC rats had greater hepatic glycogen than PTSD rats (*p* < 0.05), but still less than control (*p* < 0.05). Hepatic glycogen was similar in IHC and PTSD+IHC rats.

**Figure 5:**
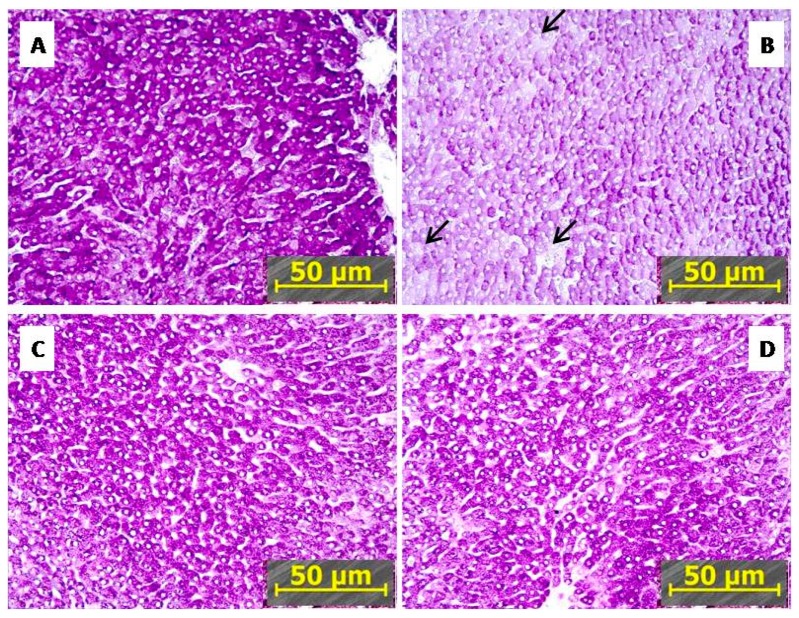
Representative microscopy of glycogen in the rat liver (Chic reaction). (**A**) control, (**B**) PTSD; (**C**) IHC, (**D**) PTSD+IHC. Arrows indicate cells deprived of glycogen. Magnification ×200.

Figure 5 shows that the staining of glycogen in sections of liver tissue from PTSD rats (Figure 5B) was considerably less intensive than in the control (Figure 5A). The glycogen reaction was less intensive and similar in liver of IHC (Figure 5C) and PTSD+IHC rats (Figure 5D) than in the control but significantly more intensive than in the liver of PTSD rats (Figure 5B). In control, IHC, and PTSD+IHC groups, glycogen granules merged into homogenous, dark masses, whereas in PTSD, glycogen granules were scarcer, diffusely spread across the cytoplasm, and were absent in some hepatocytes (Figure 5B, arrows). Although IHC alone reduced the amount of glycogen in the liver, it alleviated hepatic glycogen depletion following PTSD, as shown in both Figure 1 and Figure 5.

#### 2.3.2. IHC Attenuated PTSD-Mediated Increases in Aspartate Aminotransferase (AST) and Alanine Aminotransferase (ALT) in Rat Liver and Blood

**Figure 6:**
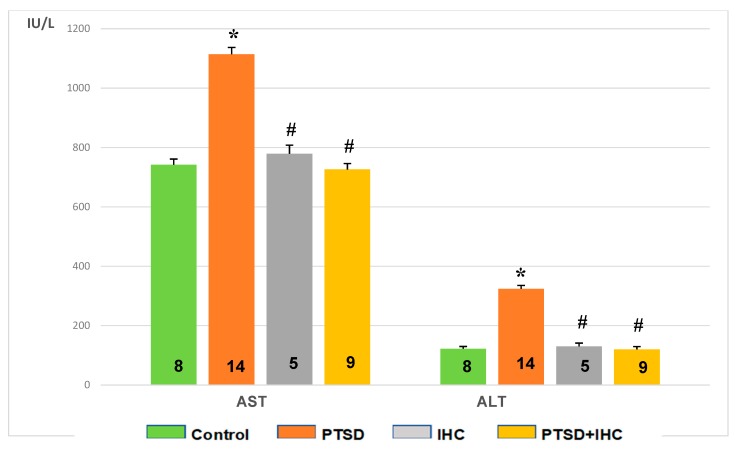
Effects of PTSD, IHC, and PTSD+IHC on activities of aspartate aminotransferase (AST) and alanine aminotransferase (ALT) in rat liver. PTSD = posttraumatic stress disorder; IHC = intermittent hypoxic conditioning. Data are presented as means ± SEM. * Significantly different from control; ^#^ Significantly different from PTSD. Numbers of rats in the groups are shown on the bars. Specific *p* values are stated in the text.

As shown in Figure 6, AST and ALT activities were increased markedly (*p* < 0.001 for both) in the liver of PTSD rats. Compared to control values, IHC did not significantly change these aminotransferase activities. In PTSD+IHC rats, IHC alleviated the stress-induced activation of both aminotransferases (*p* < 0.001), so that their activities did not differ significantly from the unstressed control values.

**Figure 7:**
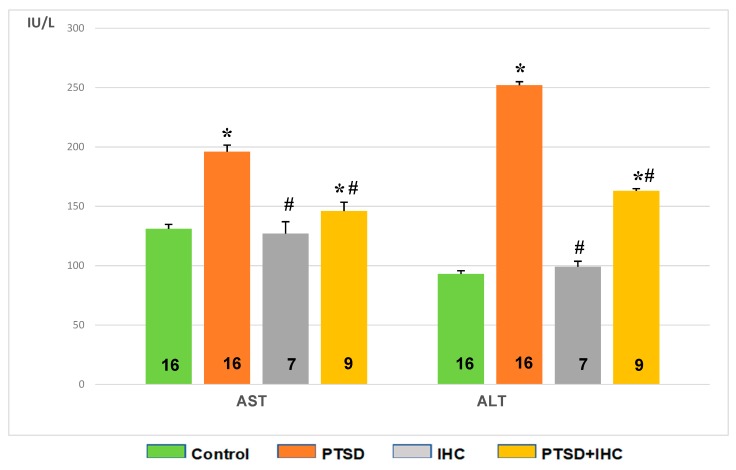
Effects of PTSD, IHC, and PTSD+IHC on activities of serum aspartate aminotransferase (AST) and alanine aminotransferase (ALT). PTSD = posttraumatic stress disorder; IHC = intermittent hypoxic conditioning. Data are presented as means ± SEM. Number of rats in groups is shown on the bars. * Significantly different from control; # significantly different from PTSD. Specific *p* values are stated in the text.

Figure 7 shows that PTSD increased activities of both AST and ALT in blood serum compared to control (*p* < 0.001). IHC did not significantly affect AST or ALT activity compared to control, but it alleviated the stress-induced increase in both enzyme activities as shown in the PTSD+IHC group (*p* < 0.001). However, these activities in PTSD+IHC rats were higher than in the unstressed control group.

#### 2.3.3. IHC Alleviated Oxidative Stress Induced by PTSD in the Liver

**Figure 8:**
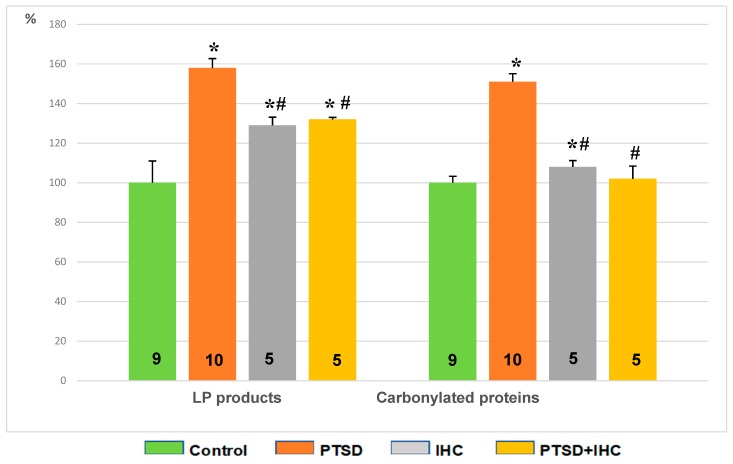
Effects of PTSD, IHC, and PTSD+IHC on concentrations of oxidative stress markers in the rat liver. PTSD = posttraumatic stress disorder; IHC = intermittent hypoxic conditioning; LP = lipid peroxidation; data are presented as means ± SEM in per cent of control values (for control, LP products = 0.213 ± 0.07 oxidation conventional units; carbonylated proteins = 273 ± 27 oxidation conventional units). Numbers of rats in the groups are shown on the bars. * Significantly different from control; ^#^ significantly different from PTSD. Specific *p* values are stated in the text.

Figure 8 shows that PTSD increased lipid peroxidation (LP) products and carbonylated proteins, i.e., protein oxidation products, compared to control (*p* < 0.001). IHC alone increased LP products (*p* = 0.006) and left unchanged the content of carbonylated proteins. IHC significantly limited oxidative stress in the liver, as evident from a 17% decrease in LP products (*p* = 0.018) and 41% decrease (*p* < 0.001) in carbonylated proteins in the PTSD+IHC rats compared to the PTSD rats. The content of LP products in PTSD+IHC rats remained significantly higher than in the control group, whereas the content of carbonylated proteins did not significantly differ from the control values.

### 2.4. IHC Prevented Detrimental Effects of PTSD on the Brain

#### 2.4.1. IHC Attenuated PTSD-Mediated Increase in NE Concentration and Decrease in MAO-A Activity

In the cerebral cortex, the NE concentration was 68% higher in PTSD rats (509 ± 46 ng/µg) than in control (301 ± 58 ng/µg, *p* = 0.014). In IHC rats, the cortical NE concentration (339 ± 53 ng/µg) did not significantly differ from control (*p* = 0.647). In the PTSD+IHC group, the NE concentration (286 ± 35 ng/µg) was lower by 60% compared to the PTSD group (*p* = 0.007) and similar to the control value. 

PTSD decreased MAO-A activity in the cerebral cortex compared to the control (4.1 ± 1.8 *vs*. 10.3 ± 3.3 nM serotonin/mg protein/min, respectively, *p* < 0.001). IHC alone caused a 32% increase in MAO-A activity (14.8 ± 1.5 nM serotonin/mg protein/min, *p* < 0.001 compared to control). In PTSD+IHC rats, the MAO-A activity was 83% higher (7.5 ± 1.1 nM serotonin/mg protein/min) than in the PTSD group; it although remained significantly lower than in control.

#### 2.4.2. IHC Alleviated Oxidative Stress Induced by PTSD in the Brain

**Figure 9:**
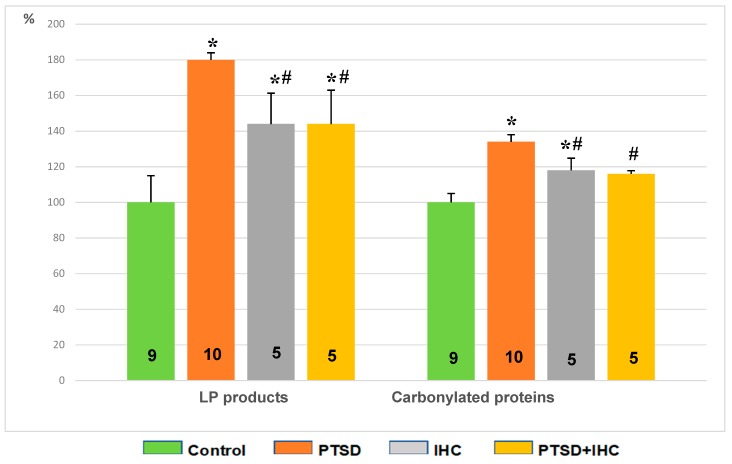
Effects of PTSD, IHC, and PTSD+IHC on concentrations of oxidative stress markers in the rat cerebral cortex. PTSD = posttraumatic stress disorder; IHC = intermittent hypoxic conditioning; LP = lipid peroxidation; data are presented as means ± SEM in per cent of control values (for control, LP products = 0.160 ± 0.07 oxidation conventional units; carbonylated proteins = 310 ± 45 oxidation conventional units). Numbers of rats in the groups are shown on the bars. * Significantly different from control; ^#^ significantly different from PTSD. Specific *p* values are stated in the text.

In rat cerebral cortex (Figure 9), PTSD increased LP products by 73% and carbonylated proteins by 34% compared to the control (*p* < 0.001 for both). IHC alone increased LP by 42% compared to the control (*p* < 0.001) and left unchanged the content of carbonylated proteins. IHC significantly restricted the PTSD-induced increase in oxidative stress; in the PTSD+IHC group, LP products were reduced by 18% (*p* = 0.002) and carbonylated proteins were reduced by 14% (*p* = 0.01), although both indexes remained significantly higher than in the control group.

## 3. Discussion

PTSD is known to be associated with both mental disorders and considerable physical comorbidity, including cardiovascular, gastrointestinal, respiratory, musculoskeletal, renal, and autoimmune diseases [20,21,22,23,24]. New PTSD comorbidities have been discovered frequently, and each of these comorbidities requires a specific treatment. At the present time, possibilities for preventing PTSD-associated comorbidities are very limited. The major objectives of this study were to identify specific PTSD-induced damages to the rat heart, liver, and brain and then to evaluate the possibility of protecting these organs with IHC, a non-pharmacological approach. Results showed that IHC prevented PTSD-associated morphological injuries in the heart and liver. IHC also alleviated depletion of hepatic glycogen, increases in activities of the injury markers, AST and ALT, in blood and liver, and also reduced disorders of norepinephrine metabolism in the cerebral cortex.

Exposure of rats to predator stress is a model of human PTSD [25]. As expected, these PTSD rats had increased anxiety-like behavior in the elevated X-maze test that resulted in a computed AI significantly higher than of control rats. In stressed PTSD+IHC rats, AI was decreased compared to that of PTSD rats, i.e., IHC produced significant protection from PTSD. This result confirms our earlier study [18]. In that earlier study, we performed the X-maze test to demonstrate that PTSD did not develop in the IHC rats exposed to predator stress in contrast to non-IHC rats exposed to the same stress.

PTSD has been shown to positively correlate with a number of cardiovascular diseases, including coronary heart disease, myocardial ischemia, and infarction [24,26,27,28], hypertension [29], tachycardia [29], and stroke [30]. PTSD is an independent risk factor for CVDs [27,28,31] and increases the risk of sudden cardiac death [32,33].

Our histological findings agree with Reznik’s prior description of PTSD-mediated ischemic injury of the myocardium of PTSD rats [34]. Loss of myocardial cross-striation was primarily due to focal I-disk destruction, as has been observed in early human myocardial infarction [19]. In Figure 3, ischemic myocardial damage was visible on polarized photographs as A disks merging into a solid, white-lightened conglomerates, specifically indicating focal myofibrillar disaggregation and lysis. In an earlier study, using light microscopy and hematoxylin-eosin staining or staining for glycogen, we observed chromatin homogenization in cardiomyocyte nuclei, interstitial edema, and decreased glycogen content in hearts of PTSD rats [35]. IHC in absence of stress produced no signs of myocardial injury, and it completely prevented the PTSD-associated morphological alterations. IHC in the absence of stress produced no signs of myocardial injury, and it completely prevented the PTSD-associated morphological alterations.

Previously, Liu et al. [36] found morphological changes characteristic of apoptosis in the myocardium of PTSD rats. These changes included chromatin pyknosis, with chromatin clotted around the nuclear membrane or dispersed in the nucleoplasm. Myocardial fibers were also ruptured, disorganized, and infiltrated by inflammatory cells. The authors suggested that the mechanism of PTSD-induced myocardial damage involved endoplasmic reticulum stress. Yuan et al. [37] reported that IHC prevented cardiac dysfunction and apoptosis by suppressing endoplasmic reticulum stress in ischemic myocardium. Data obtained by Wang and Si [38] showed that IHC protected myocardium from reperfusion injury by inhibiting myocardial apoptosis.

Histological signs of heart injury seen in PTSD rats were not observed in PTSD+IHC. While IHC cardioprotection likely involves multiple mechanisms, a role of IHC preventing myocardial oxidative stress has been well documented [11]. In the present study, we showed that PTSD potentiated oxidative stress in the myocardium, which was evident from increased concentrations of LP products and carbonylated proteins. IHC reduced these markers of oxidative stress.

An additional, although indirect marker for ischemic damage to the heart, was the elevated activities of hepatic aminotransferases, AST and ALT, in the blood, since, previously, a clinical study [39] reported that elevated AST and ALT were inversely associated with subclinical myocardial damage and impaired function. This was indicated by elevated highly sensitive cardiac troponin T and n-terminal pro-brain natriuretic peptide. Also, elevated AST and ALT are considered unfavorable prognostic factors for patients with myocardial infarction as they reflect more severe myocardial damage and dysfunction [40,41,42].

PTSD-induced increased transaminases in liver tissue generally indicate a liver injury, even in the absence of symptoms [43]. Severe acute or chronic psychological stress [44,45,46,47] has been shown to be associated with increased ALT and AST activities and hepatic inflammation [47]. Patients who developed PTSD following acute myocardial infarction had significantly higher plasma levels of ALT, AST, and alkaline phosphatase, and PTSD severity was a strong predictor for transaminase level [44]. Moreover, severe stress is associated with development of non-alcoholic fatty liver disease and ballooning degeneration of hepatocytes [48,49].

Glucose is stored as glycogen in the cytoplasm of liver and serves as the main depot source that maintains blood glucose homeostasis. During stress-induced increases in energy demand, glucagon triggers glycogen release from the liver stores and its transformation into glucose [50]. Depletion of hepatic glycogen occurs in chronic stress, including PTSD [49,51]. In humans with PTSD, the glycogen depletion may contribute to development of metabolic syndrome and its associated cardiovascular risk factors, including obesity, type 2 diabetes, dyslipidemia, and hypertension [51,52]. Survivors of the earthquake and tsunami following accident at the Fukushima Daiichi Nuclear Power Plant who developed PTSD after evacuation [53] had a significantly higher prevalence of hepatobiliary enzyme abnormality (HEA), which was defined as high ALT, AST, or GTP [53,54]. Pharmacological treatment of PTSD is likely to further damage the liver [53,55,56].

We observed that IHC significantly alleviated the stress-induced increases in AST and ALT activities and hepatic glycogen depletion. These results are consistent with data from studies showing pronounced hepatoprotective effect of direct and remote ischemic preconditioning [57], as evident from more normal activities of hepatic enzymes and reduced apoptosis of hepatocytes [58,59,60,61]. IHC, employed as in the current study, has been shown to have a hepatoprotective effect in rats exposed to high doses of alcohol [8]. The most likely mechanism of the hepatoprotection produced by hypoxic or ischemic conditioning is alleviation of oxidative stress induced by the detrimental factor due to increased activity of antioxidant enzymes [8,53,62]. The present study supports this view, since we observed reduced oxidative stress markers in PTSD+IHD rats. Another possible mechanism for the IHC-induced facilitation of glycogen accumulation in hepatocytes was proposed by Lebkova et al. [63] and Hzhehots’kyĭ et al. [62]. They suggested that during hypoxic periods, glycogen synthesis switches to using fatty acids as the primary substrate, which maintains the energy homeostasis in hepatocytes. 

Stress-induced catecholamine release is detrimental to the cerebral cortex, especially prefrontal cortex (PFC) and hippocampus [64], since high concentrations of norepinephrine (NE) impair the cortical function via α1-adrenoceptors. Thus, α1-antagonists can be protective in PTSD [65]. NE-induced damage is associated with impaired cortex cognitive function, which correlates with morphological changes, such as dendritic spine loss, dendritic atrophy, and grey matter loss in medial PFC [66,67]. Stress induced by traumatic events and resultant PTSD are recognized as a risk factor for Alzheimer’s disease [68,69]. The PTSD-induced NE accumulation results from suppressed activity and expression of monoaminoxidase A (MAO-A), a key mediator of biogenic amine metabolism [70]. Our finding of increased NE and reduced MAO-A activity in the cortex of rats with experimental PTSD are consistent with these data. A cause for suppression of MAO-A activity in PTSD is decreased levels of glucocorticoids, which are normally responsible for activation of MAO-A expression [71]. The decrease in glucocorticoids, specifically corticosterone, is, at least partially, due to dystrophy of adrenal glands as previously observed in experimental PTSD [18]. Exposure of rats to IHC significantly decreased the NE accumulation and increased the MAO-A activity in the brain cortex. These protective effects could have contributed to the lessening of anxiety behavior of PTSD+IHC rats in the X-maze. A possible mechanism for IHC prevention of the PTSD-related brain damage is alleviation of adrenal gland dystrophy and dysfunction [18], which leads to improvement of corticosterone production [18] and, thereby, improvement of MAO-A activity and restriction of NE accumulation in the cortex. 

IHC has been previously shown to exert anti-stress [8], antidepressant [72], and neuroprotective effects [15]. For example, IHC prevented stress-induced stomach ulcers [8]; the impairment of memory and brain neurodegeneration in experimental Alzheimer’s disease [12,73]; depressive anxiety-like behavior and apoptosis of brain hippocampal neurons [74] in conditions associated with cerebral damage, such as experimental Alzheimer’s disease [12,73] and chronic alcohol consumption [75]. Also, pre- and postconditioning with moderate hypobaric hypoxia prevented development of experimental PTSD in a stress-restress PTSD model [16,17]. All these detrimental conditions, including severe stress and PTSD [76], depression [77], Alzheimer’s disease [78], and chronic alcohol consumption [75], are associated with increased oxidative stress, and the authors of the studies that have demonstrated a beneficial effect of IHC suggested restriction of oxidative stress in the brain to be a major factor of IHC protection. Consistently, in the present study, experimental PTSD resulted in increased content of LP products and carbonylated proteins in the brain cortex, whereas IHC significantly decreased these oxidative stress markers, which indicated alleviation of oxidative stress as a beneficial effect of hypoxic conditioning.

In conclusion, this study has demonstrated that experimental PTSD is associated with multiorgan pathology. Results showed that the heart, liver, and brain are target organs for PTSD. IHC is a promising means for prevention of PTSD-induced damages of these target organs. The beneficial effect of IHC is at least partially due to restriction of generalized, non-specific increase in oxidative stress induced by PTSD in blood and tissues. Future clinical studies should consider IHC as a means to lessen or prevent PTSD-induced comorbidities.

## 4. Materials and Methods

### 4.1. Experimental Animals

Experiments were performed on male Wistar rats, weighing 210–230 g at the beginning of the study. Eighty rats were randomly divided into four groups of 20 each: (1) Control, (2) PTSD, (3) IHC, and (4) PTSD+IHC. Rats were housed in standard rat cages and received rat chow and tap water ad libitum. The animals were kept at controlled temperature (22–25 °C) and humidity (55%). A 12:12 h light–dark cycle was maintained with lights on between 07:00 and 19:00. All animal procedures were performed in accordance with the U.S. National Research Council Guide for the Care and Use of Laboratory Animals (publication 85-23, revised 2011). The experimental protocols (Project 0520-2019-0030) were approved by the Animal Care and Use Committee of the Institute of General Pathology and Pathophysiology (18 January 2019).

### 4.2. Modeling PTSD

To induce PTSD, we used a modified model of predator stress that was initially described by Cohen and Zohar [79] and as used in our prior studies [18,80]. Predator stress was accomplished by exposing rats of the PTSD group to cat urine scent for 15 min daily for 10 days. PTSD rats were then were given 15 days rest under predator stress-free conditions. Control rats were rested during this 25-day period. A graphical timeline of the protocol has been published [18].

### 4.3. Intermittent Hypoxia Conditioning (IHC)

IHC and PTSD+IHC rats were conditioned together in an altitude chamber for 15 consecutive days according to the previously described protocol [18]. For PTSD+IHC rats, IHC was begun on the day following completion of 10 days exposure to predator stress, and for IHC rats, IHC was begun of the 11th day of the protocol. On the 1st day of IHC, rats were exposed to an altitude of 1000 m (barometric pressure, 680 mm Hg; partial O_2_ pressure, 140 mm Hg) for 30 min; on the 2nd day—2000 m (barometric pressure, 600 mm Hg; partial O_2_ pressure, 125 mm Hg) for 1 h; on the 3rd day—3000 m (barometric pressure, 530 mm Hg; partial O_2_ pressure, 110 mm Hg) for 1.5 h; on the 4th day—4000 m (barometric pressure, 460 mm Hg; partial O_2_ pressure, 98 mm Hg) for 2 h; on the 5th day—4000 m for 3 h; and on the 6–14th days—4000 m for 4 h. The rate of ‘‘elevation” to the simulated altitude did not exceed 15 m/s. This protocol was selected for its previously demonstrated anti-stress [8,74,81], neuro- [12,14,73,81], vaso-, and cardioprotective [11,12] efficacy with no significant side effects. 

### 4.4. Behavioral Testing

The predator stress outcome was evaluated using the elevated X-maze test as employed in our prior study [18]. This test was done on the day following completion of the post-stress, 15-day rest period (PTSD group), or after completion of IHC (IHC and PTSD+IHC groups). The total duration of the test was 10 min. Control rats were tested together with rats from experimental groups in a blinded fashion. The behavior of rats in the maze was recorded and tracked using the video system SMART and analyzed with SMART 3.0 software. The number of entries into open and closed arms and the time spent in the open and closed arm were recorded. Based on these measurements, an anxiety index (AI) was calculated [82]:

AI = 1 − {[(time in open arms/Σ time on maze) + (number of entries into open arms/Σnumber of all entries)]/2}.(1)

### 4.5. Blood and Tissue Collection and Storage

Rats were sacrificed by overdose of diethyl ether, decapitated, and blood was collected. Blood serum was removed from clotted blood and stored frozen in Eppendorf tubes at −70 °C. The heart, liver, and brain cortex were excised and stored in 10% buffered formalin for histochemical analysis or frozen in liquid nitrogen and stored at −70 °C for biochemical studies.

### 4.6. Measurement of Norepinephrine

Concentration of norepinephrine (NE) was measured in the cerebral cortex. Tissue wet weight was determined, and the tissue was homogenized in 0.1 M perchloric acid. After homogenization, the samples were centrifuged (7000× *g* for 15 min at 4 °C) and the supernatants were filtered through a syringe filter (0.2 micron pore size; Whatman, Marlborough, MA, USA) before HPLC analysis on Shimadzu LC-20 Prominence Chromatographic System. HPLC analysis was performed on a C18 reversed-phase column BDS Hypersil (250 × 4.6 mm, particle sz. 5 µm) under isocratic conditions, with electrochemical detection. The mobile phase consisted of a 75 mM phosphate buffer containing 2 mM citrate acid, 0.1 mM octanesulfonic acid, and 15% (*v*/*v*) acetonitrile (pH 4.6). Electrochemical detection (DECADE II, Antc Scientific, Zoeterwoude, The Netherlands) was achieved by setting a glassy carbon working electrode at +780 mV. The final concentration of NE was expressed as ng/µg wet tissue, using an external calibration curve.

### 4.7. Measurement of MAO-A Activity

The activity of brain MAO-A was measured in brain mitochondrial fraction as described by Tipton et al. [83]. Reagents were purchased from Sigma (Basel, Switzerland). Before adding a specific MAO-A substrate, 5-hydroxytriptamine creatinine sulfate (4 mM), brain homogenates were preincubated with 100 µL of 0.5 µM selegeline, a selective, irreversible inhibitor of MAO-B activity for 60 min at 37 °C. For inhibition of MAO-B activity, 100 µL of 1 µM clorgyline was added to 1 mL of mitochondrial suspension containing MAO in the membrane-bound form and incubated for 60 min at 37 °C. Brain homogenate was prepared in 0.067 M sodium phosphate buffer (e.g., 1/10 *w/v*; pH 7.2) and centrifuged to isolate the mitochondria. The isolation of rat mitochondria was performed according to the method described by Satav and Katyare [84]. MAO activity was measured spectrophotometrically and expressed as nM serotonin/mg protein/min.

### 4.8. Measurement of Alanine (AST) and Aspartate Aminotransferase (ALT) Activities

AST and ALT activities were measured according to the method of Reitman and Frankel [85]. The method is based on aminotransferase catalysis of reversible transfer of aspartic acid aminogroups to α-ketoglutaric acid with formation of pyruvate. Pyruvate was measured photometrically at 505 nm using the reaction with 2,4-dinitrophenyl hydrazine in alkaline medium. AST and ALT were measured in blood serum and liver tissue using Klini-test-AST and Klini-test ALT, respectively, manufactured by ECO SERVICE (St. Petersburg, Russia).

### 4.9. Histochemical Analysis

Samples of heart and liver tissue were fixed in 10% buffered formalin, embedded in paraffin, and sliced into transversal 5–7 µm sections. Liver and some heart sections were used for glycogen detection, and the other heart sections were used for polarized light microscopy.

For glycogen detection, the heart and liver sections were stained with the periodic acid Schiff (PAS) reagent, which is specific for neutral muscins, and with alcian blue, which is specific for acidic muscins [86,87]. Glycogen-depleted sections were used as control. Glycogen stores in the tissue were depleted prior to the PAS reaction by incubation of deparaffinized sections with 0.46% amylase solution at 37 °C for 20 min. Optical density of section staining was measured on microphotographs with an Axioplan 2 Imaging microscope (Carl Zeiss, Berlin, Germany) using AxioVision (Carl Zeiss, Berlin, Germany) and ImageJ (NIH, Bethesda, MD, USA) software.

Deparaffinized myocardial sections were examined with polarized light microscopy (Axioplan 2 Imaging, Carl Zeiss, Berlin, Germany). Signs of myocardial ischemia was evident as white spots on dark background. Undamaged fibers remained non-luminous, i.e., black in polarized light.

### 4.10. Evaluation of Oxidative Stress

#### 4.10.1. Measurement of Lipid Peroxidation Products

The tissue content of lipid peroxidation products was assayed by an extraction, spectrophotometric method [88]. This method allows differential measurement of acyl peroxides among phospholipids extracted from propanol-2 phases along with non-esterified intermediates of fatty acid peroxides extracted from the heptane-phase. Results were expressed as oxidation indices: E232/220 for relative contents of conjugated dienes, E278/220 for ketodienes and conjugated trienes, and E400/200 for Schiff bases.

#### 4.10.2. Measurement of Protein Oxidation Products

Oxidation products were measured as carbonylated proteins according to the method of Levine et al. [89], which uses 2,4-dinitrophenylhydrazine as a reagent and measures the 2,4-dinitrophenylhydrazone derivative content in proteins. The carbonyl content was calculated using an extinction coefficient 22,000 M/l/cm and expressed as nmol protein carbonyls per mg of plasma protein. Protein concentration was determined by the Bradford protein assay method, using the Bio-Rad Protein Assay kit (Bio-Rad, Hercules, CA, USA).

### 4.11. Statistical Analysis

Values were calculated as means ± standard deviations (S.D.). A one-factor ANOVA with Neuman–Keulls test was used for multiple comparisons (e.g., control vs. PTSD, control vs. IHC, PTSD vs. IHC). Fisher’s exact test was used to compare numbers of rats with and without morphological signs of heart damage in different groups. *p*-values less than 0.05 were considered to be significant.

## Figures and Tables

**Table 1:** Effects of experimental posttraumatic stress disorder (PTSD), intermittent hypoxic conditioning (IHC), and their combination on behavioral indexes in elevated x-maze.

	Control (n = 20)	PTSD (n = 20)	IHC (n = 20)	PTSD+IHC (n = 20)
Number of entries into closed arms	6.4 ± 2.7	6.9 ± 2.8	7.8 ± 2.6	7.0 ± 2.5
Number of entries into open arms	3.3 ± 1.7	3.1 ± 1.6	4.2 ± 2.1	3.3 ± 0.7
Time spent in closed arms (s)	525.4 ± 28.3	570.0 ± 19.6 ***	488 ± 72.8 *	537 ± 15.2 ^###^
Time spent in open arms (s)	75.6 ± 28.3	30.0 ± 19.6 ***	112 ± 72.8 *	62.7 ± 15.7 ^###^
Anxiety index	0.76 ± 0.073	0.83 ± 0.051 *	0.73 ± 0.11	0.78 ± 0.043 ^#^

Data are presented as mean ± SD. Significantly different from control: * *p* < 0.05, *** *p* < 0.001; significantly different from PTSD: ^#^ *p* < 0.05; ^###^ *p* < 0.001.

## Abbreviations

PTSD	Posttraumatic stress disorder
IHC	Intermittent hypoxia conditioning
AST	Aspartate aminotransferase
ALT	Alanine aminotransferase
NE	Norepinephrine
MAO	Monoaminoxidase
